# Association between dietary inflammatory index and risk of endometriosis: A population-based analysis

**DOI:** 10.3389/fnut.2023.1077915

**Published:** 2023-02-27

**Authors:** Penglin Liu, Rashmi Maharjan, Yixiao Wang, Yubo Zhang, Yanqin Zhang, Chunyu Xu, Yuning Geng, Jinwei Miao

**Affiliations:** Department of Gynecologic Oncology, Beijing Obstetrics and Gynecology Hospital, Capital Medical University, Beijing Maternal and Child Health Care Hospital, Beijing, China

**Keywords:** endometriosis, dietary inflammatory index, diet, inflammation, National Health and Nutrition Examination Survey

## Abstract

**Background and aims:**

Chronic inflammation plays a significant role in the etiology of endometriosis, which might be affected by dietary intake. This study aimed to investigate the association between dietary inflammatory index (DII) and the risk of endometriosis.

**Methods:**

A cross-sectional analysis using data from the National Health and Nutrition Examination Survey (1999–2006) was conducted on 3,410 American participants, among whom 265 reported a diagnosis of endometriosis. DII scores were calculated based on the dietary questionnaire. The association of DII scores with endometriosis was evaluated by adjusted multivariate logistic regression analyzes, which were further investigated in the subgroups.

**Results:**

In the fully adjusted models, the odds ratio (OR) for endometriosis participants in the highest and middle tertiles of DII scores were 1.57 [95% confidence interval (CI): 1.14–2.17] and 1.18 (95% CI: 0.84–1.65), compared to the lowest tertile (*P*_trend_ = 0.007). In subgroup analyzes, the significant positive association between DII scores and the endometriosis risk was also observed in non-obese women (OR_tertile3vs1_: 1.69, 95% CI: 1.12–2.55; *P*_trend_ = 0.012), women without diabetes (OR_tertile3vs1_: 1.62, 95% CI: 1.16–2.27; *P*_trend_ = 0.005), women with hypertension (OR_tertile3vs1_: 2.25, 95% CI: 1.31–3.87; *P*_trend_ = 0.003), parous women (OR_tertile3vs1_: 1.55, 95% CI: 1.11–2.17; *P*_trend_ = 0.011), and women using oral contraceptives (OR_tertile3vs1_: 1.63, 95% CI: 1.15–2.30; *P*_trend_ = 0.006).

**Conclusion:**

This nationally representative study found that increased intake of the pro-inflammatory diet, as a higher DII score, was positively associated with endometriosis risk among American adults. Our results suggested anti-inflammatory dietary interventions may be promising in the prevention of endometriosis. Further prospective studies are necessary to confirm these findings.

## 1. Introduction

Endometriosis, a common benign gynecologic disease condition, is characterized by the implantation and growth of endometrial tissue outside the uterine cavity, causing inflammation and leading to the formation of scars and adhesion, which leads to kinds of symptoms, such as chronic pelvic pain, dysmenorrhea, dyspareunia, dysuria, and infertility (1). The disease affects women of reproductive age with a high prevalence of 5–10% (2, 3). However, current interventions for endometriosis have limited efficacy with high rates of symptom recurrence and are correlated with tremendous healthcare economic costs for long-term management (4).

Regarded as a chronic, inflammatory, endocrine, immunological, systemic and heterogeneous disease, the etiology of endometriosis remains largely elusive. However, chronic inflammation has been proposed as a well-established facilitator of the pathophysiological mechanism (1, 3). Over the past few years, extensive scientific studies have tried to identify modifiable risk factors related to endometriosis, such as exercise and diet (5). Diet, as a complex entity of anti-inflammatory or pro-inflammatory compounds, plays a critical role in modulating systemic inflammation (6–8).

The dietary inflammatory index (DII) (9), a literature-derived dietary assessment tool based on 45 food parameters, is designed to estimate the overall dietary inflammatory potential by a scoring algorithm and is associated with various inflammatory markers, including C-reactive protein (CRP), interleukin-6 (IL-6) and tumor necrosis factor-alpha (TNF-α) (10–13). Furthermore, higher DII scores, representing a stronger pro-inflammatory diet, have been verified to be related to a variety of inflammatory-related conditions including diabetes (14), chronic kidney diseases (15), cancers (16), and cardiovascular diseases (17). However, previous studies have focused on the relationship between endometriosis and specific individual nutrient intake (18–24). To our best knowledge, the relationship between DII and endometriosis has not been evaluated up to now.

Our study aims to provide the first data on the relationship between DII and the risk of endometriosis by using data from the National Health and Nutrition Examination Survey (NHANES) (25), a large population-based study in the United States (US). We also further detected whether the association varied according to obesity status, diabetes, hypertension, fertility status and the use of oral contraceptives among endometriosis patients.

## 2. Materials and methods

### 2.1. Data source

The study used a nationally representative sample from the NHANES, a cross-sectional series of interviews as well as physical and laboratory examinations conducted on the non-institutionalized US population (25). Aiming to accurately estimate the nutritional and health status of the US population over time, NHANES collects data from approximately 10,000 people on 2-year cycles, based on a complex multistage probability sampling design with a nationally representative sample. All the NHANES protocols were approved by the US National Center for Health Statistics Ethics Review Board, and written informed consent was obtained from all participants (25).

### 2.2. Sample and population

**Figure 1:**
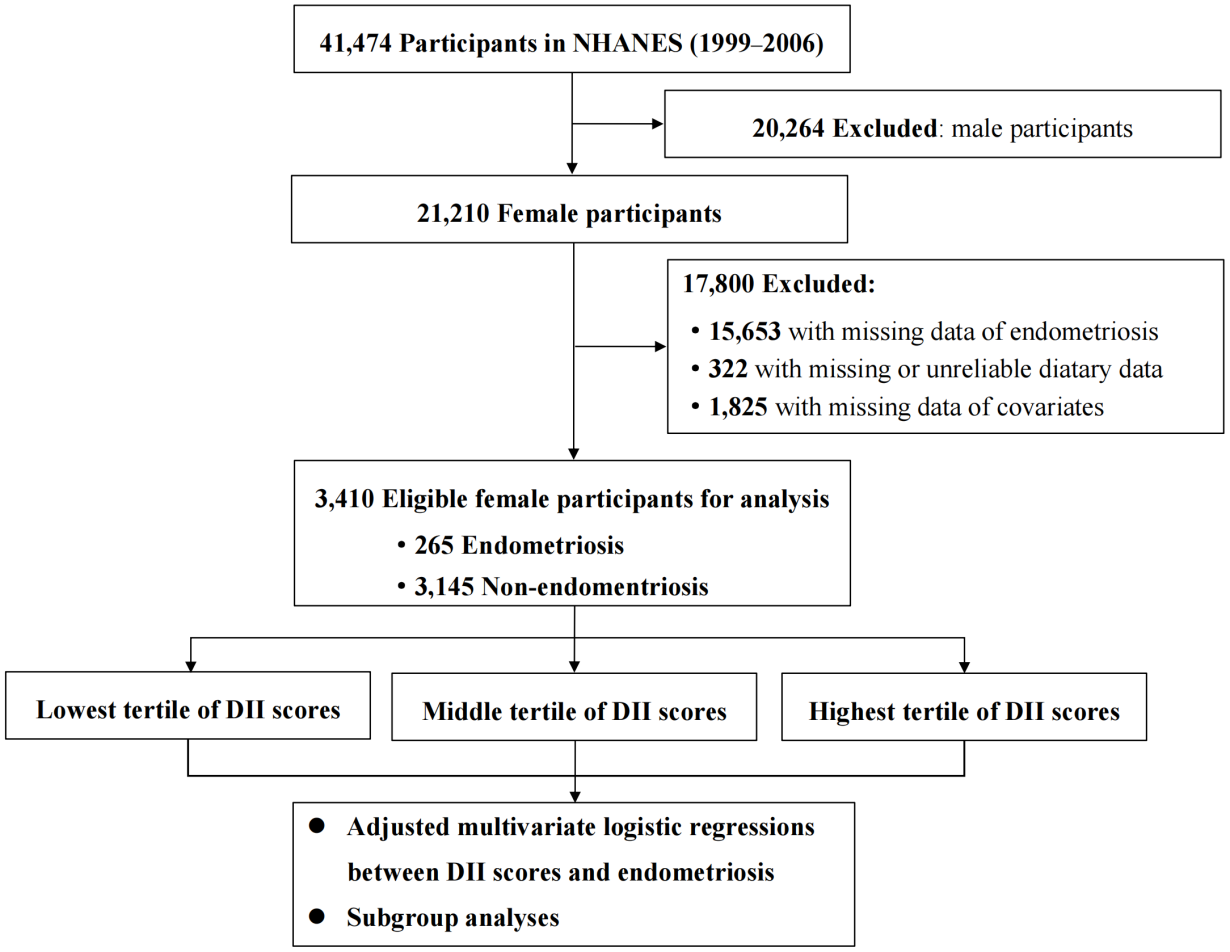
The flowchart of this study. NHANES, National Health and Nutrition Examination Survey; DII, dietary inflammatory index.

For the current study, data from 4 cycles of NHANES (1999–2000, 2001–2002, 2003–2004 and 2005–2006) were collected. A total of 21,210 female participants were enrolled, among whom women were excluded if they had missing data for the diagnosis of endometriosis (*n* = 15,653), and missing or unreliable dietary interview data (*n* = 322). In addition, those with incomplete data on the potential covariates (described below) were also excluded (*n* = 1,825). After exclusions, a total of 3,410 female participants were available for final analysis. The flowchart of the sample selection and study design is presented in Figure 1.

### 2.3. Assessment of endometriosis

NHANES included data related to the diagnosis of endometriosis in the part of the “Questionnaire on Reproductive Health” from 1999 to 2006. Participants who had endometriosis were identified if they reported “yes” to the question “Told by the doctor having endometriosis?” at each survey cycle.

### 2.4. Dietary inflammation index

The DII is a literature-derived scoring algorithm based on the data of dietary intake, designed to estimate the overall inflammatory potential of diet. The complete description of development and validation regarding the DII has been discussed in detail elsewhere (9).

Dietary data were obtained from 24-h dietary recall interviews in the part of the “Dietary Interview—Total Nutrient Intakes” and “Questionnaire on Alcohol Use,” which was utilized to calculate DII scores for all participants. DII scores of NHANES (1999–2002) were calculated based on the value of the first-day dietary interview data, since only the first-day dietary interview was conducted from 1999 to 2002. DII scores of NHANES (2003–2006) were calculated based on the mean value of the first-and second-day dietary interview data.

The DII food parameters available in the NHANES database included energy, total fat, dietary fiber, protein, carbohydrates, saturated fatty acids, monounsaturated fatty acids, polyunsaturated fatty acids, omega 3, omega 6, cholesterol, vitamin A, vitamins B1, vitamins B2, vitamin B6, vitamin B12, vitamin C, vitamin D, vitamin E, β-carotene, niacin, folic acid, Mg, Fe, Zn, Se, alcohol and caffeine. First, the Z-score of each food parameter for each participant was calculated. Second, each individual Z-score was converted to a centered percentile. Next, each centered percentile was multiplied by the standardized overall inflammatory effect score. Finally, all of the food-parameter-specific DII scores were summed to create the total DII score for each participant. The final DII score in the study was a continuous value, ranging from 4.812 (the most pro-inflammatory diet score) to -4.827 (the most anti-inflammatory diet score).

### 2.5. Covariates

According to the previous studies (26, 27) and clinical experience, the following covariates were included:

1. Demographic data: age (years), ethnicity (Mexican Americans, non-Hispanic Black, non-Hispanic White, other race), educational level (less than high school, high school graduate, above high school), marital status (married, never married, other);
2. Examination data: body mass index (BMI) was calculated as weight in kilograms divided by the square of height in meters (kg/m^2^), and obesity was identified according to the BMI (obesity: BMI ≥30 kg/m^2^, non-obesity: BMI <30 kg/m^2^);
3. Questionnaire data: drinking status (non-drinker, former drinker, current drinker), smoking status (non-smoker, former smoker, current smoker);
4. Reproductive health data: status of parity (nulliparous or ≥ one birth), use of oral contraceptives (yes or no);
5. Comprehensive data: hypertension (yes or no), diabetes (yes or no);

Hypertension was defined according to the following criteria: being told hypertension by a doctor, use of anti-hypertensive medication, the mean value of measured diastolic blood pressure ≥ 90 mmHg or the mean value of measured systolic pressure ≥ 140 mmHg (The diastolic reading with zero is not used to calculate the diastolic average; if all diastolic readings were zero, then the average would be zero; if only one blood pressure reading was obtained, that reading is the average and if there is more than one blood pressure reading, the first reading is always excluded from the average).

Diabetes was defined according to the following criteria: being told diabetes by a doctor, glycated hemoglobin A1c ≥6.5%, fasting glucose ≥7.0 mmol/l, random blood glucose ≥11.1 mmol/l, 2-h oral glucose tolerance test blood glucose ≥11.1 mmol/l, use of diabetes medication or use of insulin.

### 2.6. Statistical analysis

All analyzes were conducted following the NHANES analytic guidelines. The distribution of categorical covariates between endometriosis and non-endometriosis groups was compared by the chi-square test. The distribution of continuous variables was compared by the Wilcoxon rank sum nonparametric test or t-test according to the results of the normality test.

DII scores were categorized into three tertiles according to the distribution, and subsequent analyzes were performed. Multivariate logistic regression models were applied to estimate odd ratios (OR) and 95% confidence intervals (CI) for the associations between DII scores and endometriosis, using the lowest tertile of DII scores as the reference category. We started by fitting a crude model with only DII scores, and then further adopted 3 adjusted models. Model 1 was adjusted for age, ethnicity, education level and obesity. Model 2 included the covariates of model 1 with additional adjustment for drinking status, smoking status, diabetes and hypertension. Model 3 included the covariates of model 2 with additional adjustment for marital status, fertility status and use of oral contraceptives. Linear trends across tertiles of DII scores were examined by modeling the median value in each tertile as a continuous variable in regression models. In addition, the association between the DII scores and endometriosis risk was tested on a continuous scale by the restricted cubic spline (RCS) curve with 4 knots (at 5th, 35th, 65th, and 95th percentiles) based on the logistic regression model. Stratified analyzes were conducted by obesity (yes, no), diabetes (yes, no), hypertension (yes, no), fertility status (Nulliparous, ≥ one birth), and use of oral contraceptives (yes, no).

A *p* value <0.05 was considered statistically significant (two-sided). All statistical analyzes were performed using R software (version 4.1.3).

## 3. Results

### 3.1. Characteristics of the study sample

The distributions of baseline characteristics between endometriosis and non-endometriosis groups are presented in Table 1. From the initial sample of 21,210 female participants, 17,800 were excluded due to the incomplete data, resulting in a final sample of 3,410 eligible participants, including 265 (7.8%) endometriosis and 3,145 (92.2%) non-endometriosis women. The age of participants ranged from 20 to 54 years in the entire study. The ethnicity differed significantly between the two groups. Furthermore, participants with endometriosis tend to be older, had a higher education level, and showed a higher prevalence of drinking, smoking, using oral contraceptives, as well as hypertension.

**Table 1:** Baseline characteristics distributions between endometriosis and non-endometriosis groups.

Characteristics	Total (*n* = 3,410)	Endometriosis (*n* = 265)	Non-Endometriosis (*n* = 3,145)	*p* value
Age, median [IQR]	40.0 [32.0, 47.0]	42.0 [37.0, 48.0]	40.0 [31.0, 47.0]	<0.001
Ethnicity, *n* (%)				<0.001
Mexican American	833 (24.4)	25 (9.4)	808 (25.7)	
Non-hispanic black	798 (23.4)	45 (17.0)	753 (23.9)	
Non-hispanic white	1,494 (43.8)	180 (67.9)	1,314 (41.8)	
Other races	285 (8.4)	15 (5.7)	270 (8.6)	
Education, *n* (%)				<0.001
Less than high school	924 (27.1)	37 (14.0)	887 (28.2)	
High school graduate	831 (24.4)	80 (30.2)	751 (23.9)	
Above high school	1,655 (48.5)	148 (55.8)	1,507 (47.9)	
Obesity, *n* (%)				0.154
Yes	1,330 (39.0)	92 (34.7)	1,238 (39.4)	
No	2,080 (61.0)	173 (65.3)	1,907 (60.6)	
Smoking status, *n* (%)				0.005
Never	2,033 (59.6)	133 (50.2)	1,900 (60.4)	
Former	547 (16.0)	51 (19.2)	496 (15.8)	
Now	830 (24.3)	81 (30.6)	749 (23.8)	
Drinking status, *n* (%)				0.015
Never	557 (16.3)	28 (10.6)	529 (16.8)	
Former	570 (16.7)	54 (20.4)	516 (16.4)	
Now	2,283 (67.0)	183 (69.1)	2,100 (66.8)	
Diabetes, *n* (%)				0.179
Yes	258 (7.6)	14 (5.3)	244 (7.8)	
No	3,152 (92.4)	251 (94.7)	2,901 (92.2)	
Hypertension, *n* (%)				<0.001
Yes	941 (27.6)	104 (39.2)	837 (26.6)	
No	2,469 (72.4)	161 (60.8)	2,308 (73.4)	
Marital status, *n* (%)				0.008
Married	1987 (58.3)	168 (63.4)	1,819 (57.8)	
Never married	405 (11.9)	16 (6.0)	389 (12.4)	
Other	1,018 (29.9)	81 (30.6)	937 (29.8)	
Fertility status, *n* (%)				0.068
Nulliparous	192 (5.6)	22 (8.3)	170 (5.4)	
≥one birth	3,218 (94.4)	243 (91.7)	2,975 (94.6)	
Oral contraceptive, n (%)				<0.001
Yes	2,709 (79.4)	234 (88.3)	2,475 (78.7)	
No	701 (20.6)	31 (11.7)	670 (21.3)	
DII, median [IQR]	1.86 [0.74, 2.81]	2.11 [1.03, 3.01]	1.84 [0.71, 2.79]	0.003
DIIQ3, n (%)				0.016
Lowest tertile	1,137 (33.3)	74 (27.9)	1,063 (33.8)	
Middle tertile	1,136 (33.3)	82 (30.9)	1,054 (33.5)	
Highest tertile	1,137 (33.3)	109 (41.1)	1,028 (32.7)	

DII, dietary inflammatory index; IQR, interquartile range.

The median DII score in the entire study sample was 1.86. Cases with endometriosis have significantly higher median DII score than those without endometriosis (2.11 versus 1.84, *p* = 0.003). Moreover, a higher percentage of participants in the endometriosis group (41.1%) were found in the highest tertile of DII scores (the strongest pro-inflammatory diet) compared with 32.7% in the non-endometriosis group. The baseline characteristics of the participants according to tertile categories of DII scores are presented in Supplementary Table S1.

### 3.2. Association between DII and endometriosis

The associations between DII scores and endometriosis in the total cohort, are depicted in Table 2. The logistic regression showed that the crude OR of endometriosis was the greatest in the highest tertile of DII scores (OR 1.52, 95% CI: 1.12–2.07) compared to that in the middle (OR 1.12, 95% CI: 0.81–1.55) and lowest tertiles (*P*_trend_ = 0.008). Similarly, in adjusted multivariate models 1, 2 and 3, we identified that participants within the highest tertile of DII score were significantly associated with a higher risk of endometriosis compared to those within the lowest tertile. Furthermore, in the final fully-adjusted model (model 3) adjusted by all covariates, participants within the highest tertile of DII scores had a 57% increased risk of endometriosis compared to those within the lowest tertiles (*p* = 0.007). The trend analysis showed that the adjusted odds of endometriosis increased across increasing tertiles of DII scores in all models (Model 1: *P*_trend_ = 0.003, Model 2: *P*_trend_ = 0.007, Model 3: *P*_trend_ = 0.007).

**Table 2:** Associations between the DII score and Endometriosis among the total cohort.

DII score	Cases with endometriosis/*N*	OR (95% CI)			
		Crude^a^	Model 1^b^	Model 2^c^	Model 3^d^
Continuous values (−4.827–4.812)	265/3,410	1.14 (1.05–1.25)^*^	1.17 (1.07–1.28)^*^	1.15 (1.05–1.26)^*^	1.16 (1.06–1.26)^*^
Tertile categories					
Lowest (−4.827–1.130)	74/1,137	Reference	Reference	Reference	Reference
Middle (1.132–2.454)	82/1,136	1.12 (0.81–1.55)	1.20 (0.86–1.68)	1.18 (0.84–1.65)	1.18 (0.84–1.65)
Highest (2.458–4.812)	109/1,137	1.52 (1.12–2.07)^*^	1.62 (1.18–2.23)^*^	1.56 (1.13–2.16)^*^	1.57 (1.14–2.17)^*^
*P*_trend_		0.008	0.003	0.007	0.007

^*^Statistically significant association. ^a^Crude model was adjusted for nothing. ^b^Model 1 was adjusted for age, ethnicity, education level and obesity. ^c^Model 2 included the covariates of model 1 with additional adjustment for drinking status, smoking status, hypertension and diabetes. ^d^Model 3 included the covariates of model 2 with additional adjustment for marital status, fertility status and use of oral contraceptives. DII, dietary inflammatory index; OR, odds ratio; CI, confidence interval.

**Figure 2:**
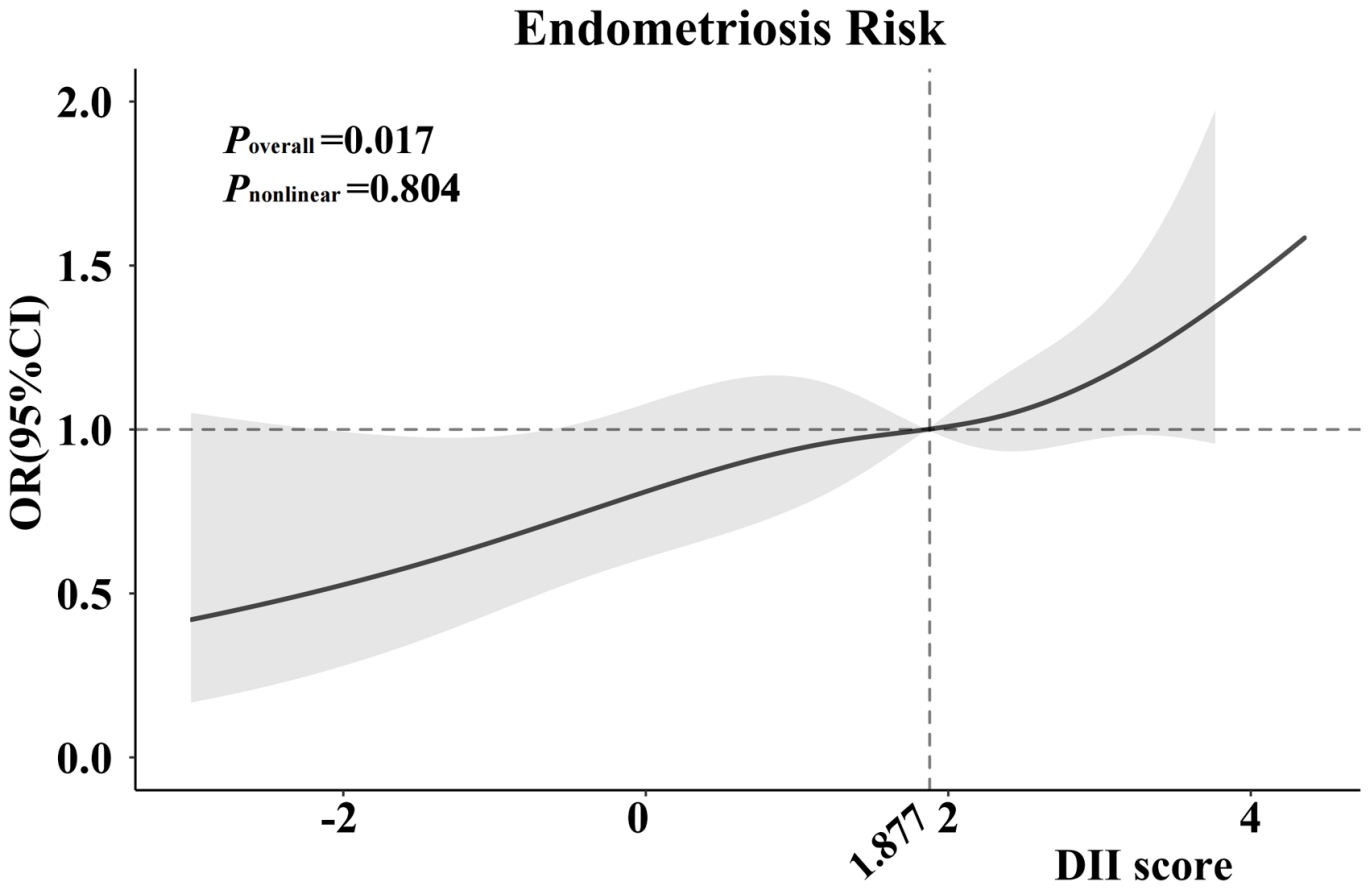
Cubic regression spline of the endometriosis risk by DII scores among the entire population. Cubic regression spline was adjusted for age, ethnicity, education level, obesity, drinking status, smoking status, hypertension, diabetes, marital status, fertility status and use of oral contraceptives. ORs are indicated by solid lines and 95% CIs are presented by shaded areas. DII, dietary inflammatory index; OR, odds ratio; CI, confidence interval.

In addition, we also detected the relationship between the DII scores and endometriosis on a continuous scale by the RCS analysis, which also showed similar results (*p* = 0.017; Figure 2).

### 3.3. Subgroup analysis

In subgroup analyzes, the positive relationship between DII scores and the risk of endometriosis differed according to the stratification of obesity, diabetes, hypertension, fertility status, and use of oral contraceptives (Table 3).

**Table 3:** Subgroup analysis of association between tertiles of DII scores and endometriosis.

Subgroup	Cases with endometriosis/N	DII scores, OR (95% CI)			
		Lowest tertile (–4.827–1.130)	Middle tertile (1.132–2.454)	Highest tertile (2.458–4.812)	*P*_trend_
Obesity
Yes	92/1,330				
Crude^a^		Reference	0.89 (0.51–1.55)	1.31 (0.78–2.17)	0.302
Model 1^b^		Reference	0.96 (0.54–1.70)	1.37 (0.81–2.33)	0.243
Model 2^c^		Reference	0.95 (0.53–1.68)	1.32 (0.77–2.25)	0.308
Model 3^d^		Reference	0.93 (0.52–1.66)	1.28 (0.75–2.20)	0.356
No	173/2,080				
Crude^a^		Reference	1.27 (0.85–1.90)	1.68 (1.14–2.47)^*^	0.009
Model 1^b^		Reference	1.36 (0.90–2.06)	1.73 (1.16–2.59)^*^	0.007
Model 2^c^		Reference	1.33 (0.88–2.02)	1.67 (1.11–2.51)^*^	0.014
Model 3^d^		Reference	1.33 (0.88–2.01)	1.69 (1.12–2.55)^*^	0.012
Diabetes
Yes	14/258				
Crude^a^		Reference	0.40 (0.08–2.03)	1.04 (0.32–3.34)	0.983
Model 1^b^		Reference	0.43 (0.08–2.33)	0.91 (0.26–3.17)	0.828
Model 2^c^		Reference	0.47 (0.08–2.75)	1.14 (0.30–4.38)	0.878
Model 3^d^		Reference	0.42 (0.07–2.65)	1.06 (0.27–4.16)	0.954
No	251/3,152				
Crude^a^		Reference	1.17 (0.84–1.64)	1.57 (1.14–2.15)^*^	0.006
Model 1^b^		Reference	1.27 (0.90–1.79)	1.68 (1.21–2.34)^*^	0.002
Model 2^c^		Reference	1.25 (0.88–1.76)	1.61 (1.15–2.26)^*^	0.005
Model 3^d^		Reference	1.24 (0.88–1.75)	1.62 (1.16–2.27)^*^	0.005
Hypertension
Yes	104/941				
Crude^a^		Reference	1.28 (0.73–2.23)	2.07 (1.24–3.44)^*^	0.005
Model 1^b^		Reference	1.37 (0.77–2.41)	2.31 (1.36–3.92)^*^	0.002
Model 2^c^		Reference	1.30 (0.73–2.31)	2.16 (1.26–3.69)^*^	0.004
Model 3^d^		Reference	1.33 (0.74–2.37)	2.25 (1.31–3.87)^*^	0.003
No	161/2,469				
Crude^a^		Reference	1.05 (0.70–1.57)	1.26 (0.85–1.86)	0.258
Model 1^b^		Reference	1.11 (0.74–1.68)	1.27 (0.84–1.91)	0.258
Model 2^c^		Reference	1.11 (0.74–1.68)	1.27 (0.84–1.91)	0.263
Model 3^d^		Reference	1.11 (0.73–1.68)	1.26 (0.83–1.91)	0.274
Fertility status
Nulliparous	22/192				
Crude^a^		Reference	1.37 (0.48–3.91)	1.19 (0.38–3.78)	0.714
Model 1^b^		Reference	1.90 (0.62–5.82)	2.05 (0.58–7.18)	0.219
Model 2^c^		Reference	1.51 (0.45–5.02)	1.34 (0.33–5.46)	0.612
Model 3^d^		Reference	1.61 (0.46–5.6)	1.13 (0.26–4.99)	0.759
≥One birth	243/3,218				
Crude^a^		Reference	1.10 (0.78–1.54)	1.56 (1.14–2.15)^*^	0.006
Model 1^b^		Reference	1.15 (0.81–1.64)	1.62 (1.16–2.26)^*^	0.004
Model 2^c^		Reference	1.14 (0.80–1.61)	1.57 (1.12–2.19)^*^	0.009
Model 3^d^		Reference	1.13 (0.79–1.60)	1.55 (1.11–2.17)^*^	0.011
Oral contraceptive	
Yes	234/2,709				
Crude^a^		Reference	1.11 (0.78–1.58)	1.53 (1.10–2.13)^*^	0.012
Model 1^b^		Reference	1.20 (0.84–1.72)	1.64 (1.16–2.31)^*^	0.005
Model 2^c^		Reference	1.19 (0.83–1.70)	1.60 (1.13–2.26)^*^	0.008
Model 3^d^		Reference	1.20 (0.83–1.72)	1.63 (1.15–2.30)^*^	0.006
No	31/701				
Crude^a^		Reference	0.99 (0.40–2.44)	1.24 (0.53–2.93)	0.637
Model 1^b^		Reference	1.23 (0.49–3.13)	1.60 (0.64–3.98)	0.317
Model 2^c^		Reference	1.03 (0.39–2.70)	1.39 (0.52–3.70)	0.533
Model 3^d^		Reference	1.03 (0.39–2.70)	1.35 (0.50–3.62)	0.572

^*^Statistically significant association. ^a^Crude model was adjusted for nothing. ^b^Model 1 was adjusted for age, ethnicity, education level and obesity. ^c^Model 2 included the covariates of model 1 with additional adjustment for drinking status, smoking status, hypertension and diabetes. ^d^Model 3 included the covariates of model 2 with additional adjustment for marital status, fertility status and use of oral contraceptives. All Models were not adjusted for the stratified covariates on which the subgroup analyzes were conducted. DII, dietary inflammatory index; OR, odds ratio; CI, confidence interval.

The final adjusted model also showed a significant positive association between tertiles of DII scores and the endometriosis risk in non-obese women (OR 1.69, 95% CI: 1.12–2.55; *p* = 0.012), in women without diabetes (OR 1.62, 95% CI: 1.16–2.27; *p* = 0.005), in women with hypertension (OR 2.25, 95% CI: 1.31–3.87; *p* = 0.003), in parous women (OR 1.55, 95% CI: 1.11–2.17; *p* = 0.011), and in women using oral contraceptives (OR 1.63, 95% CI: 1.15–2.30; *p* = 0.006). The increasing odds of endometriosis across increasing tertiles of DII scores in the above-mentioned subgroups were observed (*P*_trend_ < 0.05).

Nevertheless, no significant association was observed in obese women (OR 1.28, 95% CI: 0.75–2.20; *p* = 0.364), in women with diabetes (OR 1.06, 95% CI: 0.27–4.16; *p* = 0.933), in women without hypertension (OR 1.26, 95% CI: 0.83–1.91; *p* = 0.271), in nulliparous women (OR 1.13, 95% CI: 0.26–4.99; *p* = 0.868), and in women without the use of oral contraceptives (OR 1.35, 95% CI: 0.50–3.62; *p* = 0.549).

In addition, the magnitude of the relationship between DII scores and endometriosis was larger among women with hypertension in both crude (OR 2.07, 95% CI: 1.24–3.44) and in the fully adjusted (OR 2.25, 95% CI: 1.31–3.87) models, compared with those in the total population and other subgroups.

## 4. Discussion

### 4.1. Main finding

In this large population-based cohort from NHANES (1999–2006), a total of 3,410 participants were enrolled in the final analysis, with a 7.8% prevalence of endometriosis. We used the tool of DII score and found a significantly positive association between dietary inflammatory load and risk of endometriosis, which suggested a promising anti-inflammatory dietary intervention for the prevention of endometriosis.

### 4.2. Interpretation

To the best of our knowledge, this is the first study to determine the relationship between DII scores and the risk of endometriosis. As a common benign gynecologic disease, endometriosis exerts tremendous physical and psychological effects on the quality of life, compromises social relationships, and greatly decreases the economic productivity of society (28). Given the increasingly important role of chronic inflammation contributing to endometriosis (1, 3, 29, 30), the modifiable risk factors related to inflammatory-related conditions, including diet, have become one of the research focuses. Previous studies frequently investigated the relationship between specific dietary nutrient intake and endometriosis (18–24).

For instance, alcohol use (18), a high consumption of trans-unsaturated fat (19) and a high intake of red meat (22) were related to a higher risk of endometriosis. In contrast, a high consumption of long-chain omega 3 fatty acids (19), a high consumption of vitamin D (20), a high intake of fruits and particularly citrus fruits (21) and a high intake of dairy foods during adolescence (23) were related to a lower risk of endometriosis. The different relationships between the above-mentioned individual dietary nutrients and the risk of endometriosis might be ascribed to the different effects of their pro-inflammatory or anti-inflammatory potential. Nevertheless, the joint inflammatory effect of the total dietary nutrients on the risk of endometriosis has not been explored to date.

Based on the previous studies, our study adopted the DII score, which integrated the mixed effect of whole food parameters on inflammation (9), confirming the positive relationship between the pro-inflammatory diet and the risk of endometriosis. In this study, we observed that participants in the US general population with the highest DII scores had a 57% higher risk of endometriosis than those with the lowest DII scores after the adjustment for various covariates. Our results also support the current recommendations for a high intake of anti-inflammatory nutrients and a low intake of foods with pro-inflammatory potential.

The possible pathophysiological mechanisms of the positive relationship between DII scores and endometriosis risk might be explained by higher levels of systemic inflammation caused by the pro-inflammatory diet. Higher DII score, referring to a stronger pro-inflammatory diet, could increase the values of CRP (10, 12), IL-6 (11, 12), TNF-α (12, 13) and leucocytes as well as neutrophils (31), further contributing to the endometrial cell implantation, growth, invasion and angiogenic properties of ectopic lesions (32, 33).

Specifically, the association between the pro-inflammatory diet and the risk of endometriosis varied in subgroups in this study. When stratified by diabetes, no significant association between DII and endometriosis was observed; however, we found higher tertile of DII scores was associated with a higher risk of endometriosis among women without diabetes. Similar to endometriosis, the pathophysiology of diabetes is also closely linked to chronic inflammation triggered by the over-activation of the immune response (34–36). It is possible that women with diabetes may be bearing on a highly increased risk of endometriosis (37). Thus, the potential influence of the pro-inflammatory diet on endometriosis risk may be more impactful among women without diabetes compared with those with diabetes.

Regarding the stratified analysis by obesity, prior researches have reported a strong and consistent inverse relationship between obesity and endometriosis risk, which demonstrated that obesity might be a protective factor against endometriosis (38). In the current study, we also found that a higher DII score was significantly associated with a higher risk of endometriosis among the non-obese subgroup. Nevertheless, there was no significant relationship in the obese subgroup. This counter-intuitive result might be ascribed to the hypothesis that the role of the pro-inflammatory diet contributing to endometriosis was attenuated in the obese group due to the protective effect of obesity on endometriosis.

In the subgroup analysis stratified according to hypertension, a significant 2.25 times the risk possibility of endometriosis among women within the highest tertiles of DII scores compared with those within the lowest tertiles was observed in the hypertension subgroup. However, no significant association between DII scores and endometriosis was found among participants without hypertension. From a large prospective cohort study by Mu et al. in 2017, women with hypertension had a relatively higher risk for endometriosis (OR 1.29, 95% CI: 1.18–1.41), compared to those without hypertension (39). Similarly, our study indeed found a greater prevalence of endometriosis among patients with hypertension than those without hypertension, which was in line with the study of Mu et al. (39). Our finding implied that hypertension might enhance the ability of a pro-inflammatory diet to increase the endometriosis risk and highlighted the significance of the anti-inflammatory diet in the female population with hypertension.

Among the subgroups of nulliparous participants and those without the use of oral contraceptives, although there was a higher risk of endometriosis associated with higher tertiles of DII scores, the results were not statistically significant. Based on the previous studies, nulliparous women and women without using oral contraceptives were associated with a higher risk of endometriosis, compared to parous women and those using oral contraceptives (26, 27, 40). We speculate that the effect of the pro-inflammatory diet on endometriosis might be potentially weakened among the two specific women populations. Prospective researches are needed to perform the pro-or anti-inflammatory dietary intervention on these specific women groups to further explore the exact association between DII scores and endometriosis.

### 4.3. Strengths and limitations

The strength of this study lies in the nationally representative sample of the US population from NHANES, which allows the findings to be generalized to the total population in the US. Additionally, a wide range of potential confounding variables were adjusted in the analyzes to decrease the bias, and subgroups were performed to evaluate the association between DII scores and endometriosis in different specific populations. Moreover, to our knowledge, this study was the first to detect the association between DII scores and endometriosis, which posed a possibility of the prevention of endometriosis by an anti-inflammatory diet, contributing significantly to the reduction of health and economic burdens related to endometriosis.

Similarly, several limitations also should be considered. One limitation of this study is that the cause and effect of the association between DII scores and endometriosis could not be identified. Another limitation is that the diagnosis of endometriosis was self-reported, and was not all confirmed laparoscopically in the NHANES. Prospective randomized controlled trials are necessary to verify the association of DII with endometriosis.

## 5. Conclusion

This nationally representative study found that increased intake of the pro-inflammatory diet, as a higher DII score, was positively associated with endometriosis risk in American adults. Our findings suggested anti-inflammatory dietary interventions may be promising in the prevention of endometriosis. Further prospective studies are necessary to confirm these findings.

## Data availability statement

The original contributions presented in the study are included in the article/Supplementary material, further inquiries can be directed to the corresponding author.

## Ethics statement

The studies involving human participants were reviewed and approved by Centers for Disease Control and Prevention National Center for Health Statistics Research Ethics Review Board. The patients/participants provided their written informed consent to participate in this study.

## Author contributions

JM: conceptualization and supervision. PL and JM: data curation and writing—review and editing. RM and YW: investigation. PL and RM: methodology. PL: software, formal analysis, and writing—original draft preparation. YuZ and YaZ: visualization. CX and YG: validation. All authors contributed to the article and approved the submitted version.

## Funding

This work was supported by National Natural Science Foundation of China (grant number: 82271677); Beijing Hospitals Authority’s Ascent Plan (Code: DFL20221201); Gynecological Tumor Precise Diagnosis and Treatment Innovation Studio.

## Conflict of interest

The authors declare that the research was conducted in the absence of any commercial or financial relationships that could be construed as a potential conflict of interest.

## Publisher’s note

All claims expressed in this article are solely those of the authors and do not necessarily represent those of their affiliated organizations, or those of the publisher, the editors and the reviewers. Any product that may be evaluated in this article, or claim that may be made by its manufacturer, is not guaranteed or endorsed by the publisher.

## References

[1] Saunders PTK Horne AW. Endometriosis: etiology, pathobiology, and therapeutic prospects. Cells. (2021) 184:2807–24. doi: 10.1016/j.cell.2021.04.041, PMID: 34048704

[2] Sarria-Santamera A Orazumbekova B Terzic M Issanov A Chaowen C Asúnsolo-Del-Barco A. Systematic review and meta-analysis of incidence and prevalence of endometriosis. Healthcare. (2021) 9:29. doi: 10.3390/healthcare9010029, PMID: 33396813 PMC7824417

[3] Taylor HS Kotlyar AM Flores VA. Endometriosis is a chronic systemic disease: clinical challenges and novel innovations. Lancet. (2021) 397:839–52. doi: 10.1016/S0140-6736(21)00389-5, PMID: 33640070

[4] Simoens S Dunselman G Dirksen C Hummelshoj L Bokor A Brandes I. The burden of endometriosis: costs and quality of life of women with endometriosis and treated in referral centres. Hum Reprod. (2012) 27:1292–9. doi: 10.1093/humrep/des073, PMID: 22422778

[5] Parazzini F Viganò P Candiani M Fedele L. Diet and endometriosis risk: a literature review. Reprod Biomed Online. (2013) 26:323–36. doi: 10.1016/j.rbmo.2012.12.011, PMID: 23419794

[6] Aleksandrova K Koelman L Rodrigues CE. Dietary patterns and biomarkers of oxidative stress and inflammation: a systematic review of observational and intervention studies. Redox Biol. (2021) 42:101869. doi: 10.1016/j.redox.2021.101869, PMID: 33541846 PMC8113044

[7] Bordoni A Danesi F Dardevet D Dupont D Fernandez AS Gille D. Dairy products and inflammation: a review of the clinical evidence. Crit Rev Food Sci Nutr. (2017) 57:2497–525. doi: 10.1080/10408398.2014.967385 26287637

[8] Giugliano D Ceriello A Esposito K. The effects of diet on inflammation: emphasis on the metabolic syndrome. J Am Coll Cardiol. (2006) 48:677–85. doi: 10.1016/j.jacc.2006.03.052 16904534

[9] Shivappa N Steck SE Hurley TG Hussey JR Hébert JR. Designing and developing a literature-derived, population-based dietary inflammatory index. Public Health Nutr. (2014) 17:1689–96. doi: 10.1017/S1368980013002115, PMID: 23941862 PMC3925198

[10] Shivappa N Steck SE Hurley TG Hussey JR Ma Y Ockene IS. A population-based dietary inflammatory index predicts levels of C-reactive protein in the seasonal variation of blood cholesterol study (SEASONS). Public Health Nutr. (2014) 17:1825–33. doi: 10.1017/S1368980013002565, PMID: 24107546 PMC3983179

[11] Shivappa N Hébert JR Rietzschel ER De Buyzere ML Langlois M Debruyne E. Associations between dietary inflammatory index and inflammatory markers in the Asklepios study. Br J Nutr. (2015) 113:665–71. doi: 10.1017/S000711451400395X, PMID: 25639781 PMC4355619

[12] Tabung FK Steck SE Zhang J Ma Y Liese AD Agalliu I. Construct validation of the dietary inflammatory index among postmenopausal women. Ann Epidemiol. (2015) 25:398–405. doi: 10.1016/j.annepidem.2015.03.009, PMID: 25900255 PMC4433562

[13] Shivappa N Hebert JR Marcos A Diaz L-E Gomez S Nova E. Association between dietary inflammatory index and inflammatory markers in the HELENA study. Mol Nutr Food Res. (2017) 61:61. doi: 10.1002/mnfr.201600707, PMID: 27981781 PMC5517083

[14] Denova-Gutiérrez E Muñoz-Aguirre P Shivappa N Hébert JR Tolentino-Mayo L Batis C. Dietary inflammatory index and type 2 diabetes mellitus in adults: the diabetes mellitus survey of Mexico City. Nutrients. (2018) 10:385. doi: 10.3390/nu10040385, PMID: 29561774 PMC5946170

[15] Mazidi M Shivappa N Wirth MD Hebert JR Kengne AP. Greater dietary inflammatory index score is associated with higher likelihood of chronic kidney disease. Br J Nutr. (2018) 120:204–9. doi: 10.1017/S0007114518001071, PMID: 29947319

[16] Fowler ME Akinyemiju TF. Meta-analysis of the association between dietary inflammatory index (DII) and cancer outcomes. Int J Cancer. (2017) 141:2215–27. doi: 10.1002/ijc.30922, PMID: 28795402 PMC6056732

[17] Tyrovolas S Koyanagi A Kotsakis GA Panagiotakos D Shivappa N Wirth MD. Dietary inflammatory potential is linked to cardiovascular disease risk burden in the US adult population. Int J Cardiol. (2017) 240:409–13. doi: 10.1016/j.ijcard.2017.04.104, PMID: 28487151 PMC5518308

[18] Matalliotakis IM Cakmak H Fragouli YG Goumenou AG Mahutte NG Arici A. Epidemiological characteristics in women with and without endometriosis in the Yale series. Arch Gynecol Obstet. (2008) 277:389–93. doi: 10.1007/s00404-007-0479-1, PMID: 17922285

[19] Missmer SA Chavarro JE Malspeis S Bertone-Johnson ER Hornstein MD Spiegelman D. A prospective study of dietary fat consumption and endometriosis risk. Hum Reprod. (2010) 25:1528–35. doi: 10.1093/humrep/deq044, PMID: 20332166 PMC2873173

[20] Harris HR Chavarro JE Malspeis S Willett WC Missmer SA. Dairy-food, calcium, magnesium, and vitamin D intake and endometriosis: a prospective cohort study. Am J Epidemiol. (2013) 177:420–30. doi: 10.1093/aje/kws247, PMID: 23380045 PMC3626048

[21] Harris HR Eke AC Chavarro JE Missmer SA. Fruit and vegetable consumption and risk of endometriosis. Hum Reprod. (2018) 33:715–27. doi: 10.1093/humrep/dey014, PMID: 29401293 PMC6018917

[22] Yamamoto A Harris HR Vitonis AF Chavarro JE Missmer SA. A prospective cohort study of meat and fish consumption and endometriosis risk. Am J Obstet Gynecol. (2018) 219:178.e1–178.e10. doi: 10.1016/j.ajog.2018.05.034, PMID: 29870739 PMC6066416

[23] Nodler JL Harris HR Chavarro JE Frazier AL Missmer SA. Dairy consumption during adolescence and endometriosis risk. Am J Obstet Gynecol. (2020) 222:257.e1–257.e16. doi: 10.1016/j.ajog.2019.09.010, PMID: 31526789 PMC7056553

[24] Schwartz NRM Afeiche MC Terry KL Farland LV Chavarro JE Missmer SA. Glycemic index, glycemic load, fiber, and gluten intake and risk of Laparoscopically confirmed endometriosis in premenopausal women. J Nutr. (2022) 152:2088–96. doi: 10.1093/jn/nxac107, PMID: 35554558 PMC9445851

[25] *Centers for Disease Control and Prevention. About the National Health and Nutrition Examination Survey*. Available at: http://www.cdc.gov/nchs/nhanes/about_nhanes.htm

[26] Vercellini P Viganò P Somigliana E Fedele L. Endometriosis: pathogenesis and treatment. Nat Rev Endocrinol. (2014) 10:261–75. doi: 10.1038/nrendo.2013.255 24366116

[27] Shafrir AL Farland LV Shah DK Harris HR Kvaskoff M Zondervan K. Risk for and consequences of endometriosis: a critical epidemiologic review. Baillieres Best Pract Res Clin Obstet Gynaecol. (2018) 51:1–15. doi: 10.1016/j.bpobgyn.2018.06.001, PMID: 30017581

[28] Della Corte L Di Filippo C Gabrielli O Reppuccia S La Rosa VL Ragusa R. The burden of endometriosis on Women's lifespan: a narrative overview on quality of life and psychosocial wellbeing. Int J Environ Res Public Health. (2020) 17:4683. doi: 10.3390/ijerph17134683, PMID: 32610665 PMC7370081

[29] Capobianco A Monno A Cottone L Venneri MA Biziato D Di Puppo F. Proangiogenic Tie2(+) macrophages infiltrate human and murine endometriotic lesions and dictate their growth in a mouse model of the disease. Am J Pathol. (2011) 179:2651–9. doi: 10.1016/j.ajpath.2011.07.029, PMID: 21924227 PMC3204092

[30] Symons LK Miller JE Kay VR Marks RM Liblik K Koti M. The Immunopathophysiology of endometriosis. Trends Mol Med. (2018) 24:748–62. doi: 10.1016/j.molmed.2018.07.004, PMID: 30054239

[31] Wirth MD Sevoyan M Hofseth L Shivappa N Hurley TG Hébert JR. The dietary inflammatory index is associated with elevated white blood cell counts in the National Health and nutrition examination survey. Brain Behav Immun. (2018) 69:296–303. doi: 10.1016/j.bbi.2017.12.003, PMID: 29217263 PMC5857420

[32] Guo F He Y Fan Y Du Z Sun H Feng Z. G-CSF and IL-6 may be involved in formation of endometriosis lesions by increasing the expression of angiogenic factors in neutrophils. Mol Hum Reprod. (2021) 27:27. doi: 10.1093/molehr/gaab064, PMID: 34643696

[33] Symons LK Miller JE Tyryshkin K Monsanto SP Marks RM Lingegowda H. Neutrophil recruitment and function in endometriosis patients and a syngeneic murine model. FASEB J. (2020) 34:1558–75. doi: 10.1096/fj.201902272R, PMID: 31914688

[34] Cabrera SM Henschel AM Hessner MJ. Innate inflammation in type 1 diabetes. Transl Res. (2016) 167:214–27. doi: 10.1016/j.trsl.2015.04.011, PMID: 25980926 PMC4626442

[35] Böni-Schnetzler M Meier DT. Islet inflammation in type 2 diabetes. Semin Immunopathol. (2019) 41:501–13. doi: 10.1007/s00281-019-00745-4, PMID: 30989320 PMC6592966

[36] Rohm TV Meier DT Olefsky JM Donath MY. Inflammation in obesity, diabetes, and related disorders. Immunity. (2022) 55:31–55. doi: 10.1016/j.immuni.2021.12.013, PMID: 35021057 PMC8773457

[37] Simmen RCM Brown DM Quick CM Alhallak I Rose TK Liu S. Co-morbidity of type 1 diabetes and endometriosis: bringing a new paradigm into focus. J Endocrinol. (2019) 243:248. doi: 10.1530/JOE-19-0248, PMID: 31472479

[38] Farland LV Missmer SA Bijon A Gusto G Gelot A Clavel-Chapelon F. Associations among body size across the life course, adult height and endometriosis. Hum Reprod. (2017) 32:1732–42. doi: 10.1093/humrep/dex207, PMID: 28591798 PMC5850750

[39] Mu F Rich-Edwards J Rimm EB Spiegelman D Forman JP Missmer SA. Association between endometriosis and hypercholesterolemia or hypertension. Hypertension. (2017) 70:59–65. doi: 10.1161/HYPERTENSIONAHA.117.09056, PMID: 28559401 PMC6469492

[40] Vercellini P Eskenazi B Consonni D Somigliana E Parazzini F Abbiati A. Oral contraceptives and risk of endometriosis: a systematic review and meta-analysis. Hum Reprod Update. (2011) 17:159–70. doi: 10.1093/humupd/dmq042, PMID: 20833638

