# Supplementary material for: Association between dietary inflammatory index and risk of endometriosis: A population-based analysis
Source: Front Nutr. 2023 Feb 27;10:1077915. doi: 10.3389/fnut.2023.1077915 (PMC10008869; doi:10.3389/fnut.2023.1077915)
Supplement: Supplementary file 1 [file Table_1.docx]

Supplementary Material

**Table S1 Baseline characteristics distributions according to tertile categories of DII scores.**

| **Characteristics** | **Total**  **(n=3,410)** | **DII score** | | | ***P* value** |
| --- | --- | --- | --- | --- | --- |
|  |  | **Lowest Tertile**  **(n=1,137)** | **Middle Tertile**  **(n=1,136)** | **Highest Tertile**  **(n=1,137)** |  |
| **Age, median [IQR]** | 40.0  [32.0, 47.0] | 41.0  [34.0, 48.0] | 40.0  [31.5, 47.0] | 39.0  [31.0, 46.0] | <0.001 |
| **Ethnicity, n (%)** |  |  |  |  | <0.001 |
| Mexican American | 833 (24.4) | 325 (28.6) | 289 (25.4) | 219 (19.3) |  |
| Non-Hispanic Black | 798 (23.4) | 203 (17.9) | 278 (24.5) | 317 (27.9) |  |
| Non-Hispanic White | 1,494 (43.8) | 494 (43.4) | 479 (42.2) | 521 (45.8) |  |
| Other Races | 285 (8.4) | 115 (10.1) | 90 (7.9) | 80 (7.0) |  |
| **Education, n (%)** |  |  |  |  | <0.001 |
| Less than high school | 924 (27.1) | 288 (25.3) | 315 (27.7) | 321 (28.2) |  |
| High school graduate | 831 (24.4) | 225 (19.8) | 266 (23.4) | 340 (29.9) |  |
| Above high school | 1,655 (48.5) | 624 (54.9) | 555 (48.9) | 476 (41.9) |  |
| **Obesity, n (%)** |  |  |  |  | 0.071 |
| Yes | 1,330 (39.0) | 415 (36.5) | 447 (39.3) | 468 (41.2) |  |
| No | 2,080 (61.0) | 722 (63.5) | 689 (60.7) | 669 (58.8) |  |
| **Smoking status, n (%)** |  |  |  |  | <0.001 |
| Never | 2,033 (59.6) | 748 (65.8) | 683 (60.1) | 602 (52.9) |  |
| Former | 547 (16.0) | 202 (17.8) | 181 (15.9) | 164 (14.4) |  |
| Now | 830 (24.3) | 187 (16.4) | 272 (23.9) | 371 (32.6) |  |
| **Drinking status, n (%)** |  |  |  |  | 0.330 |
| Never | 557 (16.3) | 187 (16.4) | 187 (16.5) | 183 (16.1) |  |
| Former | 570 (16.7) | 178 (15.7) | 180 (15.8) | 212 (18.6) |  |
| Now | 2,283 (67.0) | 772 (67.9) | 769 (67.7) | 742 (65.3) |  |
| **Diabetes, n (%)** |  |  |  |  | 0.314 |
| Yes | 258 (7.6) | 93 (8.2) | 75 (6.6) | 90 (7.9) |  |
| No | 3,152 (92.4) | 1,044 (91.8) | 1,061 (93.4) | 1,047 (92.1) |  |
| **Hypertension, n (%)** |  |  |  |  | 0.592 |
| Yes | 941 (27.6) | 315 (27.7) | 302 (26.6) | 324 (28.5) |  |
| No | 2,469 (72.4) | 822 (72.3) | 834 (73.4) | 813 (71.5) |  |
| **Marital status, n (%)** |  |  |  |  | <0.001 |
| Married | 1,987 (58.3) | 722 (63.5) | 624 (54.9) | 641 (56.4) |  |
| Never married | 405 (11.9) | 114 (10.0) | 137 (12.1) | 154 (13.5) |  |
| Other | 1,018 (29.9) | 301 (26.5) | 375 (33.0) | 342 (30.1) |  |
| **Fertility status, n (%)** |  |  |  |  | 0.164 |
| Nulliparous | 192 (5.6) | 71 (6.2) | 69 (6.1) | 52 (4.6) |  |
| ≥one birth | 3,218 (94.4) | 1,066 (93.8) | 1,067 (93.9) | 1,085 (95.4) |  |
| **Oral contraceptive, n (%)** | |  |  |  | 0.015 |
| Yes | 2,709 (79.4) | 871 (76.6) | 917 (80.7) | 921 (81.0) |  |
| No | 701 (20.6) | 266 (23.4) | 219 (19.3) | 216 (19.0) |  |

***Note****:* DII, dietary inflammatory index; IQR, interquartile range.
